# In reply: Why selinexor remains a subpar option?

**DOI:** 10.1093/oncolo/oyag113

**Published:** 2026-03-27

**Authors:** Ghulam Rehman Mohyuddin, Tomer Meirson

**Affiliations:** Division of Hematology and Hematological Malignancies, Huntsman Cancer Institute, University of Utah, Salt Lake City, UT 84106, United States; Division of Medical Oncology, Davidoff Cancer Center, Petah Tikva 4910000, Israel

We appreciate the interest of Richter et al. in our analysis of censoring in the BOSTON trial, a randomized comparison of selinexor/bortezomib/dexamethasone versus bortezomib/dexamethasone in myeloma.^1^ The authors suggest that bortezomib/dexamethasone was not an inferior comparator given that at the time the trial design was first conceived, which was presumably much earlier than when the first patient enrolled on BOSTON, the control arm of bortezomib/dexamethasone was valid. Such a logic runs afoul of all modern ethical conventions of research and ignores the ground realities of how a patient is consented.^2^ At the time a patient is consented to a research trial, the ethical practice is to discuss standard-of-care alternatives to research, and if the standard-of-care therapy is much superior to the control arm of the trial, then the control arm of a trial is unethical and should not be offered to patients. By this standard, bortezomib/dexamethasone was not an appropriate regimen for patients in the United States where the BOSTON trial enrolled patients.

Even if we contend that the control arm was appropriate, subsequent trials of novel immunotherapy drugs with much better control arms such as daratumumab/bortezomib/dexamethasone have shown superiority of these regimens,^3–5^ rendering selinexor obsolete for first relapse in today’s age, especially as these novel regimens are increasingly easily accessible in the community.

While acknowledging its utility for assessing censoring patterns, the authors dismiss the reverse Kaplan Meier analysis, arguing that digitized data approximations are inaccurate and that the sensitivity analyses lack support in regulatory science. We acknowledge that without access to individual patient-level data and follow-up for each of those patients, we cannot infer with certainty whether informative censoring would have invalidated the results of this trial. The authors do not deny that unbalanced attrition and withdrawal from the triplet arm occurred, nor do they aim to provide reasons for why such attrition occurred. To validate the reconstruction methodology, we compared our estimated HR (0.66) to the reported HR (0.70); the 0.04 difference aligns with the accuracy benchmarks utilized in prior reconstructions.^6^ Although this small discrepancy stems from our unstratified approach, the triplet arm notably lost statistical significance in the sensitivity analysis despite the favorable HR. While the authors highlight the reconstruction discrepancies, these uncertainties could be more effectively resolved through the provision of individual patient data, allowing for a definitive assessment of the sensitivity analysis.

The high rate of early discontinuation in the BOSTON trial is not a mere artifact of digital reconstruction; the published life table demonstrates a 10% excess in censoring within the triplet arm by 7 months. This threshold of imbalance has drawn significant regulatory scrutiny. In the quizartinib registration trial in acute myeloid leukemia, for example, early censoring was frequent, with over 10% excess censoring observed in the control arm.^7^ Similarly, in the PANORAMA-1 trial of panobinostat in multiple myeloma, high dropout rate with excess censoring in the triplet arm also exceeded 10%.^8^ In both instances, excessive censoring was central to the FDA convening the Oncologic Drugs Advisory Committee (ODAC) to question whether substantial differences in early censoring, rather than a true treatment effect, generated the statistically significant benefit.

The authors provide an analysis of response rates comparing censored versus uncensored patients, noting that the former had a higher response rate , which they argue predicts favorable progression free survival (PFS) However, this comparison conflates administrative censoring with informative censoring. Most censoring events in a trial occur at data lock; these administratively censored patients are, by definition, those who survived without progression until the end of the study window. Consequently, it is expected that the aggregate “censored” group would demonstrate higher response rates. This obscures the outcomes of the specific subgroup of concern: those censored early due to informative reasons, such as frailty. To truly assess bias, one would need to compare the rates of patients censored early against the remaining cohort. However, the authors face a paradox in attempting to use surrogate endpoints like response rate or time to next treatment to exonerate these early dropouts: to definitively establish that these patients had superior outcomes, they must be followed, yet they were censored. If their outcomes were observable enough to confirm benefit, they arguably should not have been censored for the primary endpoint. Furthermore, using time to next treatment as an indicator for a reliable PFS analysis—without providing the associated survival curves or details regarding censoring—is unhelpful. Such an analysis is susceptible to the similar selection biases, representing only a comparison of the subset of patients who remained in active follow-up.

The FDA explicitly encourages the provision of sensitivity analyses to evaluate the robustness of trial results, particularly regarding the impact of missing data and the sensitivity of observed outcomes to various statistical assumptions.^9^^,^^10^ Similarly, guidelines published by the Common Sense Oncology initiative advocate for sensitivity analyses that include a “worst-case” assessment, assuming patients progressed at the time of censoring^11^—an approach also employed by the FDA, including during the ODAC review of the aforementioned PANORAMA-1 trial.^8^ We show that the PFS benefit in the BOSTON trial is sensitive to the fate of the excess censored patients; specifically, the statistical significance of the result is lost if we assume that these patients had worse outcomes.

It is also important to note that all reported subsequent randomized trials of selinexor in myeloma have been unsuccessful in meeting statistical significance for their primary endpoint, including a randomized trial with identical design to the BOSTON trial run in China,^12^ and a trial of selinexor and lenalidomide versus lenalidomide alone in the post-transplant maintenance setting.^13^ This raises concern that the original finding seen in BOSTON was a spurious one that was inflated by informative censoring, given that the clinical benefit of selinexor has not yet been reproduced in a subsequent randomized trial.

Ultimately, our opinion is that each patient offered selinexor at first relapse in 2026 and beyond is a patient who could have been meaningfully offered a more effective treatment option. As a result, we maintain that allowing subpar options to remain on NCCN guidelines represents an ultimate disservice to patients and could potentially erode outcomes.
